# Fungi That Live Within Animals: Application of Cell Cytometry to Examine Fungal Colonization of Ambrosia Beetle (*Xyleborus* sp.) Mycangia

**DOI:** 10.3390/jof11030184

**Published:** 2025-02-26

**Authors:** Ross A. Joseph, Kamaldeep Bansal, Jane Nguyen, Michael Bielanski, Esther Tirmizi, Abolfazl Masoudi, Nemat O. Keyhani

**Affiliations:** 1 Department of Biological Sciences, University of Illinois at Chicago, Chicago, IL 60607, USA; etirm2@uic.edu (E.T.); amasou7@uic.edu (A.M.); 2 USDA-ARS-Subtropical Horticulture Research Station, Miami, FL 33158, USA; kamaldeep.bansal@usda.gov; 3 Department of Microbiology and Cell Science, University of Florida, Gainesville, FL 32611, USA; jtn40@med.miami.edu (J.N.); m.bielanski@ufl.edu (M.B.)

**Keywords:** fungal mutualism, ambrosia beetles, *Xyleborus*, mycangia, cell cytometry, *Harringtonia* and *Raffaelea* sp.

## Abstract

Ambrosia beetles bore into trees, excavating galleries where they farm fungi as their sole source of nutrition. These mutualistic fungi typically do not cause significant damage to host trees; however, since their invasion into the U.S., the beetle *Xyleborus glabratus* has vectored its fungal partner, *Harringtonia lauricola*, which has acted as a devastating plant pathogen resulting in the deaths of over 500 million trees. Here, we show differences in the mycangial colonization of the indigenous *X. affinis* ambrosia beetle by *H. lauricola*, and the native fungal species, *H. aguacate* and *Raffaelea arxii*. While *X. affinis* was a good host for *H. lauricola*, the related ambrosia beetle, *X. ferrugineus*, was only marginally colonized by *H. lauricola*. *X. affinis* beetles neither fed on, nor were colonized by, the distantly related fungus, *Magnaporthe oryzae*. Mycangial colonization was affected by the nutritional state of the fungus. A novel method for direct quantification of mycangial contents based on image cell cytometry was developed and validated. The method was used to confirm mycangial colonization and demonstrate alternating fungal partner switching, which showed significant variation and dynamic turnover. *X. affinis* pre-oral mycangial pouches were visualized using fluorescent and light microscopy, revealing that newly emerged pupae displayed uncolonized mycangia prior to feeding, whereas beetles fed *H. lauricola* contained single-celled fungi within 6 h post-feeding. Mixed populations of fungal cells were seen in the mycangia of beetles following alternating colonization. Nuclear counter-staining revealed insect cells surrounding the mycangia. These data highlight variation and specificity in ambrosia beetle–fungal pairings and provide a facile method for direct quantification of mycangial contents.

## 1. Introduction

Fungal mutualisms with animals have existed for over 300 million years, with significant ecological and evolutionary consequences, as exemplified by diverse lineages of beetles (ambrosia beetles), which have independently “domesticated” equally diverse lineages of obligate fungal partners as their major, if not sole, sources of nutrition [1,2,3]. Thus, the term “ambrosia beetle”, refers to a polyphyletic group of tree- and wood-boring weevils (*Curculionidae: Platypodinae* and *Scolytinae*) which have convergently evolved fungal “farming” with similarly diverse lineages of fungi (e.g., select members within the *Hypocreaceae*, *Ophiostomataceae*, *Polyporales*, and *Ceratocystidaceae*). Ambrosia beetles excavate galleries in host tree sapwood where they cultivate their fungal partners [4]. In some instances, the beetle–fungus partnership is apparently specific, whereas for others, the beetle can associate with a variety of closely related fungal partner species, sometimes having more than one partner at the same time [5,6,7,8,9]. Xyleborini ambrosia beetles associate with a consortium that can include facultative bacteria and yeasts but typically have at least one major obligate filamentous fungal partner. Ambrosia beetles inoculate the wood substrate of gallery walls with fungal spores during excavation, growing nutritionally enriched cells for beetle consumption [10,11].

Most ambrosia beetle–fungal pairings do not result in the death of host trees. However, there are several instances of ambrosia fungi, vectored by their beetle partner, acting as phytopathogens, particularly those that are invasive [2,12]. Around the turn of the 21st century, an invasive beetle to the southeastern United States, *Xyleborus glabratus*, harboring its fungal symbiont, since named *Harringtonia lauricola*, has led to an epidemic disease of lauraceous plants, termed laurel wilt, that has killed hundreds of millions of redbay, swamp bay, sassafras trees, and now threatens the avocado industry in the United States [13,14,15]. *H. lauricola* has been shown to be the causative agent of laurel wilt and its potential spread westwards to California and south to Central and South America is of significant concern. *H. lauricola* has now been isolated from environmental samples of indigenous *Xyleborus* beetles, and the ability of these beetles to maintain *H. lauricola* as their major symbiont has been confirmed in the laboratory [13,16,17]. Although various physiological, secondary metabolite syntheses and stress responses of *H. lauricola* have been characterized [18,19,20], the mechanism(s) mediating plant pathogenicity remain obscure. Fungal cells are thought to proliferate in the plant xylem, causing damage and potentially a series of hypersensitive responses, which are ultimately lethal to the plant [21].

In ambrosia beetles, the fungal inoculum is maintained in specialized insect transport structures termed mycangia, which sustain the mutualism between microbe and host into new environments and across beetle generations [22,23]. However, since fungal domestication has independently evolved in different beetle lineages, there is a wide diversity of mycangial shapes, sizes, locations on the insect body, and even distribution between the sexes [24,25]. These structures include ectodermal/exoskeletal invaginations, e.g., pits and shallow bowls, as well as tubes, cavities, and pouches within the insect body, some of which are lined with hairs and/or fed by glandular cells. Complicating matters, in some instances, clearly defined mycangia have not been found [26]. However, methods for interrogating the contents and/or dynamics of mycangial colonization by partner fungi remain sparse. *Xyleborus* (female) ambrosia beetles have twin pre-oral mycangia located within the head, just beneath the mandibles [17]. Structural studies of preoral paired mycangia from several other genera of beetles, including *Euwallacea*, *Ambrosiophilus*, *Ambrosiodmus*, and *Premnobius* have been performed [6,24,27,28,29]. Micro-CT scanning across the life stages of *Euwallacea validus* revealed the absence of mycangia in larvae and early-stage pupae, while these structures could be detected in late-stage pupae and adult females [24]. *Xyleborus* beetles can harbor fungal species now separated into the *Raffaelea*, *Harringtonia*, and *Dryadomyces* genera (all previously characterized as *Raffaelea*). These different species can co-occur within the same beetle/beetle colony, indicating flexibility regarding the fungal partner association [8,30,31].

More recently, the pre-oral mycangia of *Xyleborus* beetles were probed using fungal strains transformed to express reporter (eGFP and RFP)-marker genes [17]. These previous data showed that colonization followed a characteristic time course and was stable to starvation. Transmission electron microscopy showed a dimorphic shift in the growth of the fungus in the mycangia, although the characteristics of these cells remain obscure. Here, we expand upon these results to provide new methodologies and insights into the physiology and cellular mechanisms by which the symbiotic association within the pre-oral mycangia of *Xyleborus* beetles function.

## 2. Materials and Methods

### 2.1. Insect Rearing and Fungal Strains and Culture Conditions

*Xyleborus affinis* and *Xyleborus ferrugineus* beetles were collected by light trapping in Gainesville, Florida during the spring and summer seasons (2021–2023). Following sunset, a white sheet was stretched between two posts and bright white light was shone on it to attract insects. Ethanol was also used as an attractant by splashing it directly onto the sheet or by hanging a 50 mL tube in front of the sheet containing cotton balls soaked in 100% ethanol [26]. Ambrosia beetles attracted to the sheet were collected into vials and identified in the laboratory using a dissecting microscope [32]. Colonies of *X. affinis* and *X. ferrugineus* were maintained under laboratory conditions, and aposymbiotic beetles were reared for colonization experiments as previously described [17]. Briefly, sawdust agar medium was made by mixing 60 g wood flour, 15 g coarse sweetgum sawdust, 20 g agar, 10 g sucrose, 5 g corn starch, 5 g casein, 5 g yeast extract, 1 g Wesson salt mixture, and 2.5 mL wheat germ oil into 500 mL of water. Following autoclaving and cooling, 350 mg streptomycin and 10 mg tetracycline were suspended in 5 mL 95% ethanol and added to the mixture. Roughly 15–20 mL of this mixture was added to 50 mL tubes and allowed to dry for at least seven days prior to initiating colonies. All reagents were obtained from Fisher Scientific (Thermo Fisher Scientific, Hampton, NH, USA) unless otherwise noted. Three days before initiating colonies, a square of agar containing actively growing *H. lauricola* was cut out of a Petri dish and placed into media tubes to inoculate them. Concurrently, the surface of the sawdust media in the culture tubes was scratched with a sterile probe to create a rough surface that was easier for beetles to begin burrowing through. To initiate colonies, 3–15 female beetles and 1–3 male beetles were added to tubes, and the tubes were maintained with caps loosely tightened for 25–30 days in the dark at 23–25 °C. Following this incubation period, the sawdust medium was removed from colony tubes and carefully dissected to remove adult beetles, pupae, and larvae. Adult beetles were used to initiate new colony tubes while the pupae and larvae were separated, and the surface was sterilized with 70% ethanol followed by three washes with sterile distilled water and maintained on sterile moistened filter paper until the adult beetles emerged to generate aposymbiotic beetles for colonization assays.

Fungal strains of wild type, green-, and red-fluorescent protein (eGFP and RFP) expressing strains of *Harringtonia lauricola* (RL4), and wild type *H. aguacate* (PL1004) and *Raffaelea arxii* (CBS273.70) were maintained as glycerol stocks at −80 °C and routinely grown and maintained on potato dextrose agar/broth (PDA/PDB), Sabouraud dextrose agar (SDA), and/or Czapek-Dox agar (CZA) [17,33]. Culture mediums were purchased from Fisher Scientific. *Magnaporthe oryzae* (KV1) was kindly provided by Dr. Jessie Fernandez for use in colonization experiments and was maintained in her lab at the University of Florida. Plates were incubated in the dark at 25 °C for 7–21 d. Colonization assays were performed as in [17] with minor modifications. Briefly, 96-well plates containing 100 μL PDA in each well were inoculated using sterile toothpicks by scraping fungal spores from mycelia on an actively growing plate and stabbing it into each well. Plates were then grown as above for 5–7 days prior to introducing beetles into wells. For colonization assays testing growth from different media types, 100 μL of each medium was pipetted into wells of a 96-well plate. *H. lauricola* spores were collected into water, the concentration was adjusted to 1 × 10^8^ spores/mL, and 1 μL of this solution was spotted onto wells containing each media type. These cultures were then grown for 7 d, as above, prior to introducing beetles into the wells.

### 2.2. Experimental Mycangia Colonization, Determination of Colony Forming Units (CFUs), and Cell Cytometry Assays

Aposymbiotic female beetles were experimentally colonized with desired strains and species of fungi by placing them into individual wells of 96-well plates containing the desired medium and previously inoculated as above. Beetles were allowed to feed on fungal cultures for the desired amount of time pertaining to the timepoints examined in the experiments, and plates were stored in the dark at 25 °C. Plates were periodically examined to ensure that beetles were not stuck on their backs or to the walls of wells and were able to freely feed on the fungal cultures. Following experimental feeding periods, beetles were removed from wells and, using two sterile syringe needles, their head (containing mycangia) was removed from the body and placed into a sterile 1.5 mL tube. The individual heads in the tubes were then surface sterilized by washing with 70% ethanol for two minutes, followed by three water washes for one minute each. After this last wash, the heads were resuspended in 200 μL of water, and a sterile glass bead was added to the tube. Beetle heads were then macerated in a bead beater (MP Fast Prep 24, MP Biomedicals, Solon, OH, USA) at 4 m/s for one minute. Following bead beating, the solution containing the macerated beetle head was diluted 5-fold into a final volume of 200 μL of water and 50 μL of this solution was plated in triplicate onto 60 mm Petri dishes containing PDA amended with 200 μg/mL cycloheximide and 100 μg/mL streptomycin (Thermo Fisher Scientific, Hampton, NH, USA) [17]. For experiments involving culturing of *Magnaporthe oryzae*, cycloheximide was not included in the media formulation as the fungus was susceptible to this selection agent. CFU plates were incubated in the dark at 25 °C until countable colonies appeared (5–7 days), at which point colonies were manually counted for each plate, and the average of the three triplicates was used as the CFU count for that beetle.

For cell cytometry analysis, heads of colonized beetles were removed, and their surfaces were sterilized as above before being fixed briefly in a 4% paraformaldehyde (PFA) solution in phosphate-buffered saline (PBS) (Electron Microscopy Sciences, Hatfield, PA, USA). PFA was then removed, and the heads were washed three times in water, suspended in 200 μL of water, and macerated by bead beating. This undiluted solution was then pipetted into individual wells of a 96-well plate. Plates were left on the benchtop for 20–30 min to allow fungal cells to settle to the bottom of the wells. Plates were then loaded into a Keyence BZ-X800 fluorescence microscope (Keyence Corporation of America, Itasca, IL, USA) with image cytometry hardware for analysis. The microscope was programmed to autofocus using the brightfield channel every capture, with 25% light intensity and 20% aperture with a 1/2500 s exposure time before switching to the RFP and GFP channels with 10% laser intensity and 1 s exposure time. Images of quadrants covering the entire area of the wells were captured to collect images of all cells present in the well. The compilation of images was then processed using the Keyence BZX-800 Analyzer software V1.1.2.4 (Keyence Corporation of America, Itasca, IL, USA) to yield a quantity of fungal cells per well representative of the number of fungal cells contained in the mycangia of each beetle.

### 2.3. Microscopy

To obtain microscope images of fungal cells within *X. affinis* mycangia, aposymbiotic beetles were colonized using GFP- and RFP-expressing *H. lauricola* as above, embedded in Optimal Cutting Temperature (OCT) mounting medium (Sakura Finetek USA, Torrance, CA, USA) and frozen in a bath of isopentane cooled in liquid nitrogen. Frozen blocks were then sectioned using a Leica 3050S cryostat (Leica, Wetzlar, Germany) to a thickness of 5–20 μm. Sections were collected directly onto microscope slides, followed by fixation in 4% PFA for 20 min and three washes in sterile distilled water for three minutes each to remove PFA and OCT. Slides were then dried and mounted in Vectashield hardset mounting medium containing DAPI (Vector laboratories, Plain City, OH, USA) for fluorescent staining of the nuclei. Mounted sections were then visualized using a Keyence BZX-800 fluorescence microscope at 40×, 60×, and 100× objective magnification using brightfield, TRITC, GFP, and DAPI fluorescent channels. For fluorescent images, Z stacks were collected over a range encompassing the signal and assembled into a full-focus image in the Keyence BZX-800 Analyzer software. For brightfield photos, single images were taken and overlayed with full-focus fluorescent images.

### 2.4. Data Analysis

All violin and box and whisker plots were generated in R (R core team, 2024, https://www.R-project.org/ (accessed on 5 January 2025)) using the GGplot2 V3.5.1 package [34]. Datapoints plotted on these graphs consisted of the averages of three technical replicates for CFU experiments and the total cell counts per beetle from image cytometry experiments. All statistical analyses were performed in R using: dplyr V1.1.4 (dplyr: A Grammar of Data Manipulation, R package version 1.1.4, https://CRAN.R-project.org/package=dplyr (accessed on 5 January 2025)), car V3.1.2 (An R Companion to Applied Regression 3rd edition. Sage, Thousand Oaks, CA, USA, https://www.john-fox.ca/Companion/index.html (accessed on 5 January 2025)), and/or FSA V0.9.6 (FSA: Simple Fisheries Stock Assessment Methods, R package version 0.9.6, https://CRAN.R-project.org/package=FSA (accessed on 5 January 2025)). The Shapiro–Wilk test assessed the normality of distribution within sample groups. Levene’s test evaluated the homogeneity of variances across groups, while the Wilcoxon rank-sum test was applied for non-parametric comparisons between two groups. For the non-parametric comparison of multiple groups, the Kruskal–Wallis test was used with post hoc pairwise comparisons made using the Dunn test with Bonferroni correction. For pairwise comparisons of normally distributed samples, *t*-tests were used to compare between groups. For multiple comparisons of normally distributed data, ANOVA tests were used with the Tukey HSD post hoc test.

## 3. Results

### 3.1. Mycangial Colonization Is Dependent upon Beetle and Fungal Partner Species

**Figure 1:**
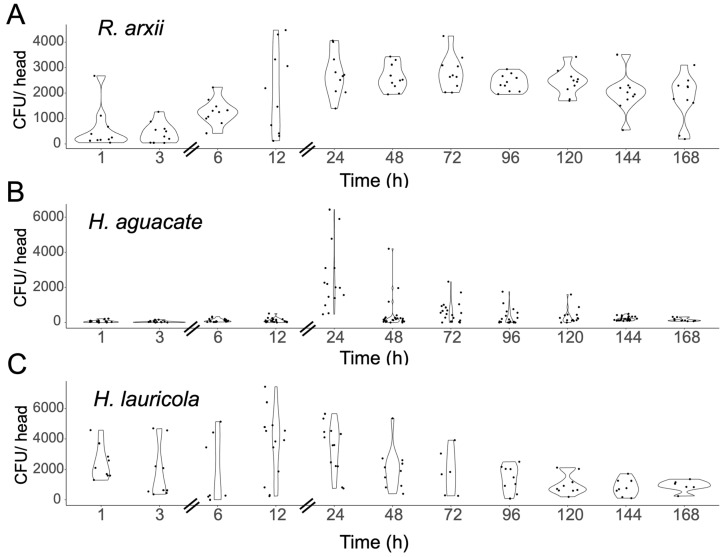
Mycangial colonization curves for *X. affinis* beetles fed on *Raffaelea arxii* (**A**), *Harringtonia aguacate* (**B**), and *Harringtonia lauricola* (**C**). Beetles were allowed to feed on fungal cultures in individual wells of a 96-well plate for 1–168 h and processed for mycangial content determination as detailed in the Methods section. Statistically significant differences between 1 h and other timepoints: (**A**) 1–24 (*p* = 0.0015), 1–48 (*p* = 0.0031), 1–72 (*p* = 0.0007), 1–96 (*p* = 0.017), and 1–120 (*p* = 0.018). (**B**) 1–24 (*p* = 0.00000001), 1–72 (*p* = 0.005), and 1–144 (*p* = 0.02). (**C**) No significance.

**Figure 2:**
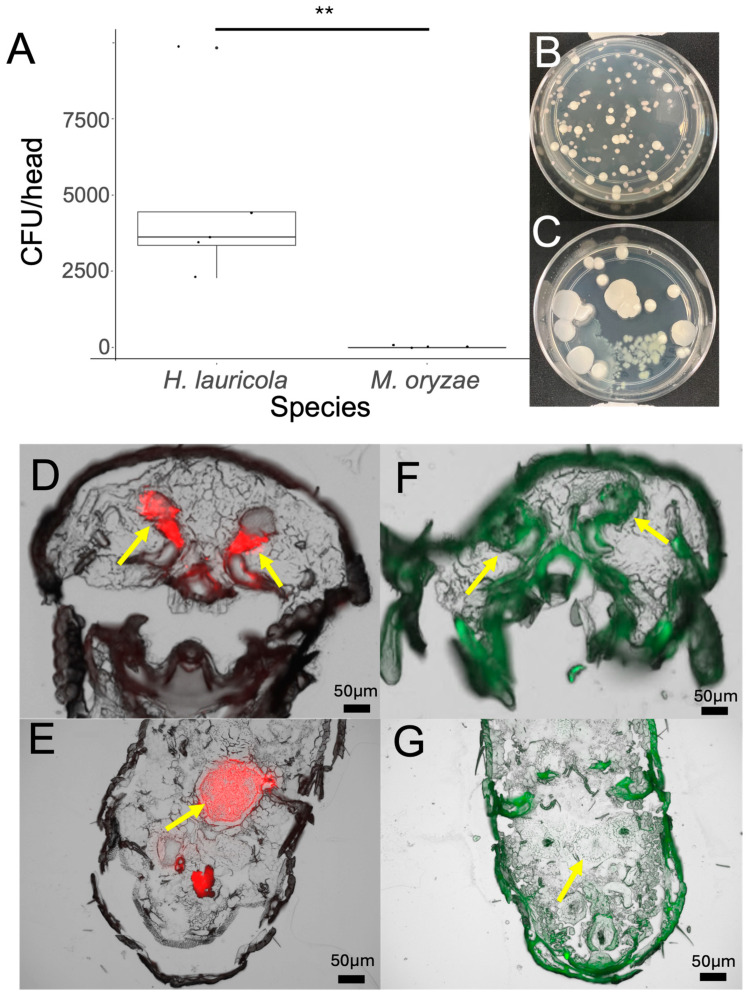
Mycangial colonization is specific. Results of allowing *X. affinis* beetles to feed on an ambrosia fungus symbiont (RFP-expressing *H. lauricola*) or a non-symbiotic plant pathogenic fungus (GFP-expressing *Magnaporthe oryzae*) for 24 h. (**A**–**C**) Quantification of mycangial cell counts and representative image of CFU plates, respectively. (**D**,**E**) Cross-sectional images of the mycangia and gut, respectively, of beetles fed *H. lauricola*. (**F**,**G**). Cross-sectional images of the mycangia and gut, respectively, of beetles fed *M. oryzae*. Asterisks in panel (**A**) indicate a *p*-value of 0.0075.

**Figure 3:**
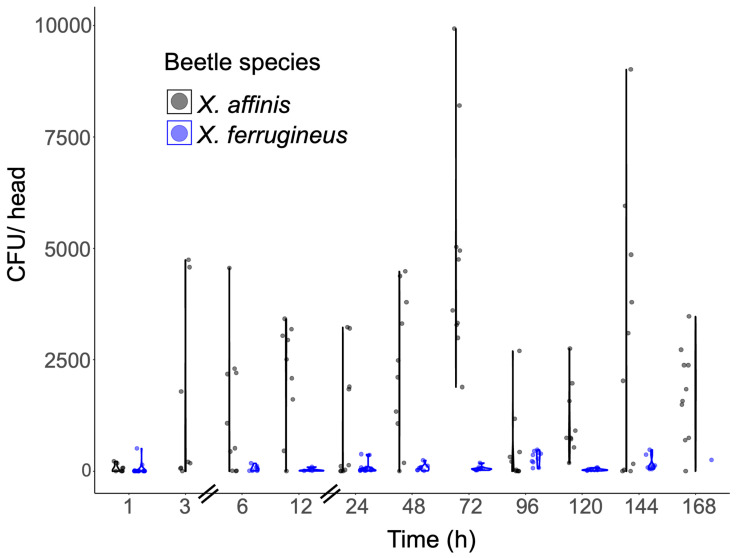
Comparison of mycangial colonization by *Harringtonia lauricola* in two ambrosia beetle species: *X. affinis* (gray) and *X. ferrugineus* (blue). All timepoints (except 3 and 168, which lack sufficient sample size to test) show significant differences between species of *p* < 0.0001.

Newly emerged aposymbiotic *X. affinis* adults were fed with different ambrosia beetle fungal partners including *Harringtonia lauricola*, *H. aguacate*, and *Raffaelea arxii*, or the non-ambrosia beetle fungus, *Magnaporthe oryzae*, a well-known plant fungal pathogen, over a time course up to 7 d (Figure 1 and Figure 2). These data indicated robust colonization by *R. arxii* and *H. lauricola* over the entire time, although some differences in the temporal dynamics between the two fungal partners were noted. As previously described, (the invasive, non-native to the US) *H. lauricola* showed rapid colonization of indigenous (to the US) *X. affinis* mycangia within 1 h of feeding, with a slight increase within the 12–24 h time period, followed by a gradual decrease to a lower steady state within the 96–168 h (4–7 d) time period examined, with a wide sample variation seen. The native ambrosia fungal species, *R. arxii*, showed a slightly more gradual colonization curve (1–24 h, peaking at 24 h), which was then maintained (from 24 to 120 h), only gradually decreasing at the later timepoints (144–168 h, 6–7 d). In contrast, (the native) *H. aguacate* showed very poor colonization of *X. affinis* mycangia, with little to no colonization until 24 h, at which timepoint, a notable wide variation in fungal cell counts recovered was seen, after which levels dropped back to very low colonization (72 h and beyond). To validate specificity of mycangial colonization, a GFP-expressing strain of the non-ambrosia beetle fungus but plant pathogen, *Magnaporthe oryzae*, was fed to newly emerged aposymbiotic *X. affinis* adults (Figure 2A–C). Whereas colonization by *H. lauricola* and accompanying fungal CFU recovery was apparent for the positive control, no fungal counts were recovered after *M. oryzae* feeding, although bacterial colonies were recovered for the latter and not the former. Sections from beetle heads as well as gut sections were examined by fluorescence microscopy with RFP (corresponding to *H. lauricola*) but not GFP (*M. oryzae*) evident in both mycangial pouches and the gut, which, for the latter, showed no signal aside from autofluorescence of insect structures (Figure 2D–G). As indicated, we have previously shown and confirmed here that *H. lauricola* can effectively colonize the mycangia of (the native) *X. affinis* beetles. To determine whether this phenomenon could be extended more broadly to other (native) *Xyleborus* species, we examined *H. lauricola* colonization of *X. ferrugineus* (Figure 3). These data showed little to no mycangial colonization of *X. ferrugineus* over the 7 d time course examined.

### 3.2. Mycangial Colonization Is Affected by the Nutritional State of the Partner Fungus, and Long-Term Switching of Partner Fungi Can Occur

**Figure 4:**
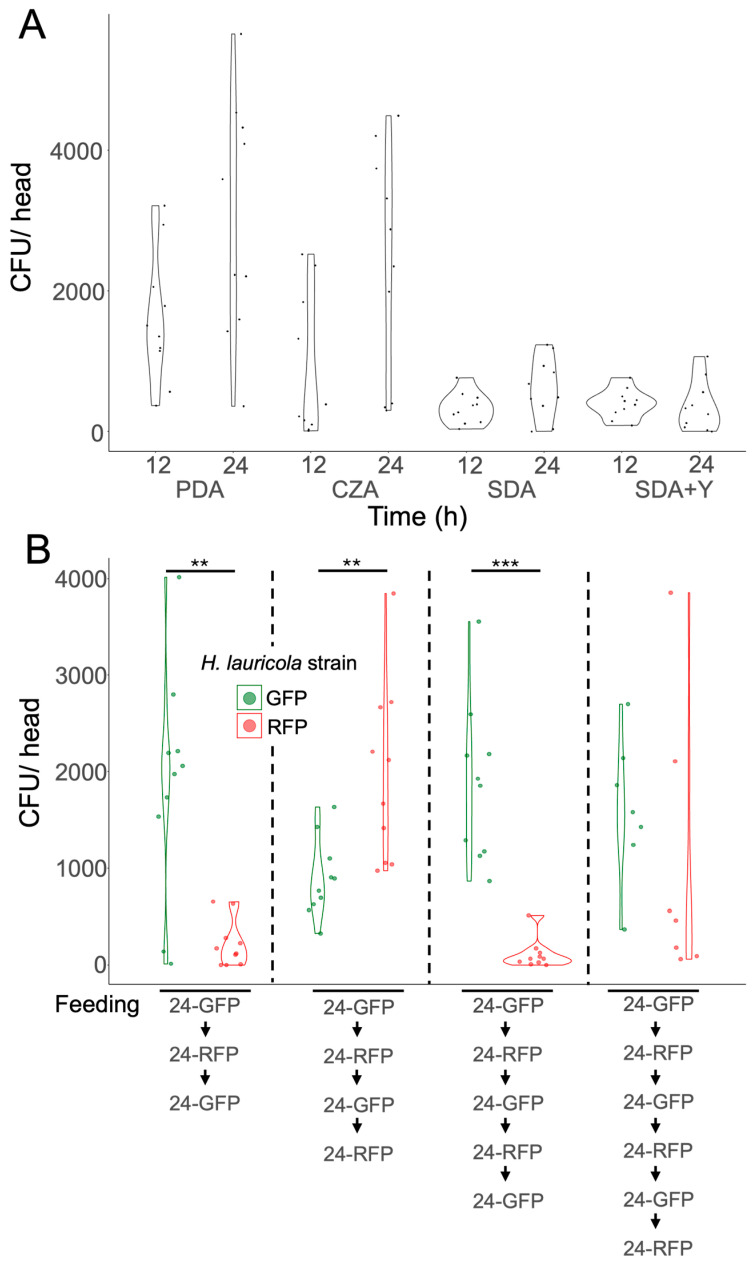
Nutritional status of the fungus affects mycangial colonization. (**A**) Mycangial colonization of *X. affinis* beetle by *H. lauricola* grown on different media as indicated, and (**B**) turnover of *H. lauricola* cells within mycangia. Media comparisons at the 12 h timepoint show significant differences: PDA vs. SDA (*p* = 0.0019), and PDA vs. SDA+Y (*p* = 0.0044). Media comparisons at the 24 h timepoint show significant differences: PDA vs. SDA (*p* = 0.00045), PDA vs. SDA+Y (*p* = 0.0001023), CZA vs. SDA (*p* = 0.0105), and CZA vs. SDA+Y (*p* = 0.0027). (**B**) Asterisks indicate *p*-values as follows: ** = 0.001 < *p* < 0.01, *** = 0.0001 < *p* < 0.001.

To examine the effects of the nutritional state of the fungal partner on efficiency of mycangial colonization, *H. lauricola* was grown on four different media: PDA (dextrose, medium nutrition), CZA (minimal media + sucrose, low nutrition), SDA (rich media), and SDA + yeast extract (enhanced rich media) before newly emerged aposymbiotic beetles (*X. affinis*) were allowed to feed for 12 and 24 h (Figure 4A). The mycangial colonization of fungal cells grown on PDA or CZA showed robust colonization at both 12 and 24 h (albeit with significant variation), but with mean levels significantly (*p* < 0.001) higher than *H. lauricola* grown on SDA or SDA+Y, which remained low.

We have previously shown that mycangial occupancy is dynamic and shows turnover under conditions of continuous feeding when the host fungal partner is switched during the initial 3 d of colonization. However, these experiments did not examine turnover once the mycangia had been fully colonized, i.e., at timepoints > 3 d, nor at timepoints in which a stable mycangial occupancy had occurred (>24 h), nor after a series of sequential switching. As before, to track fungal cell switching in the mycangia, we used two *H. lauricola* reporter strains, namely, *H. lauricola^GFP^* and *H. lauricola^RFP^*. To examine the dynamic nature of the mycangia over long-term occupancy, newly emerged *X. affinis* beetles were first exposed to/allowed to feed on *H. lauricola^GFP^* for 24 h, after which the beetles were transferred to wells containing *H. lauricola^RFP^* for 24, then back to the *H. lauricola^GFP^* for 24 h (3 d), with the sequential switching continued for 4, 5, and 6 d (Figure 4B). Beetles fed the GFP-expressing strain (for 24 h), then the RFP-expressing strain (24 h), then back to the GFP-expressing strain (24 h), showed a high level of GFP-cells (1867 ± 370), with low levels of the RFP (220 ± 77). Beetles fed GFP-RFP-GFP-RFP retained a moderate level of the GFP cells (894 ± 126), with RFP overtaking (1971 ± 294). Beetles fed GFP-RFP-GFP-RFP-GFP showed a dramatic decrease in RFP (110 ± 48), with high levels of GFP cells (1873 ± 257). Beetles fed GFP-RFP-GFP-RFP-GFP-RFP again retained moderate to high levels of GFP cells (1616 ± 278), with most beetles showing only low RFP cell levels (1045 ± 538), although significant variation was seen.

### 3.3. Application of Cell Cytometry to Quantify Mycangial Content and Imaging of the Mycangia

**Figure 5:**
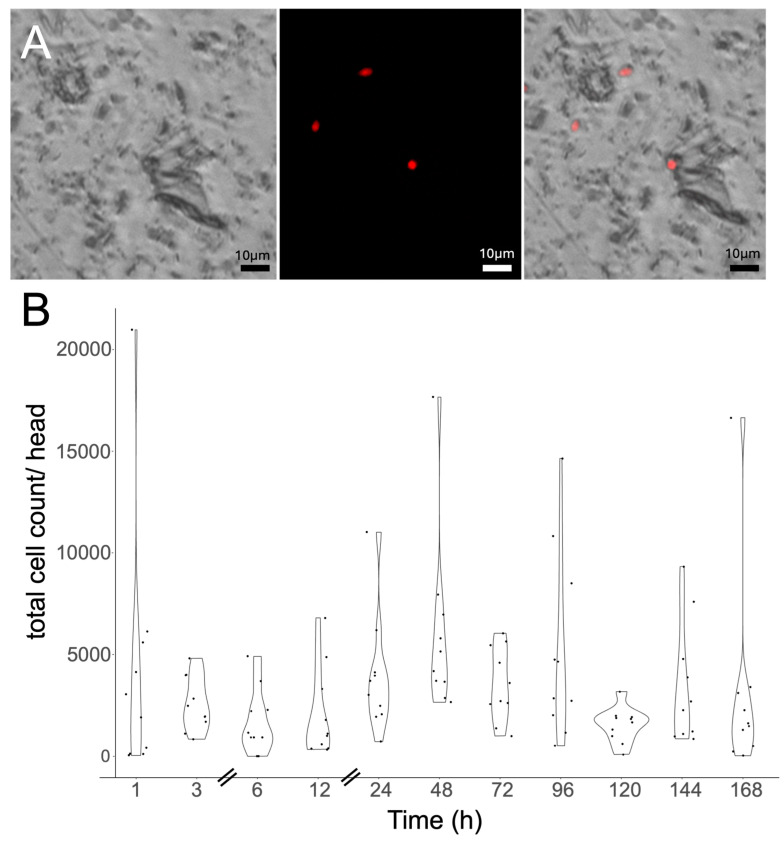
Cell image cytometry: quantification of *H. lauricola* colonization of *X. affinis* mycangia. (**A**) Representative images of RFP-expressing *H. lauricola* cells detected by image cytometry. Left image: brightfield channel, center image: RFP channel, right image: overlay. (**B**) Quantification of image cytometry results. No significant differences between the 1 h timepoint and other timepoints were noted.

To develop a more medium-throughput assay that did not rely on the plating of mycangial extract and waiting for fungal colonies to be apparent (3–4 d) and then counting (CFU approach), we applied cell cytometry to directly visualize and count cells in the mycangia. Using the *H. lauricola^RFP^* and *H. lauricola^GFP^* reporter strains, we first confirmed that fluorescent fungal cells could be visualized and counted in multi-well plates as detailed in the Methods section. These experiments indicated concentration-dependent fungal cell cytometry counting that showed >95% correspondence to direct cell counting using a hemocytometer and by CFU plating. As the cell cytometry coupled imaging of samples with cell counting, both the qualitative images and quantitative cell counts were obtained (Figure 5A). To validate the method, a time course of *H. lauricola* occupancy of newly emerged *X. affinis* beetles was performed (Figure 5B). These data were in good agreement with that collected via CFU counting.

**Figure 6:**
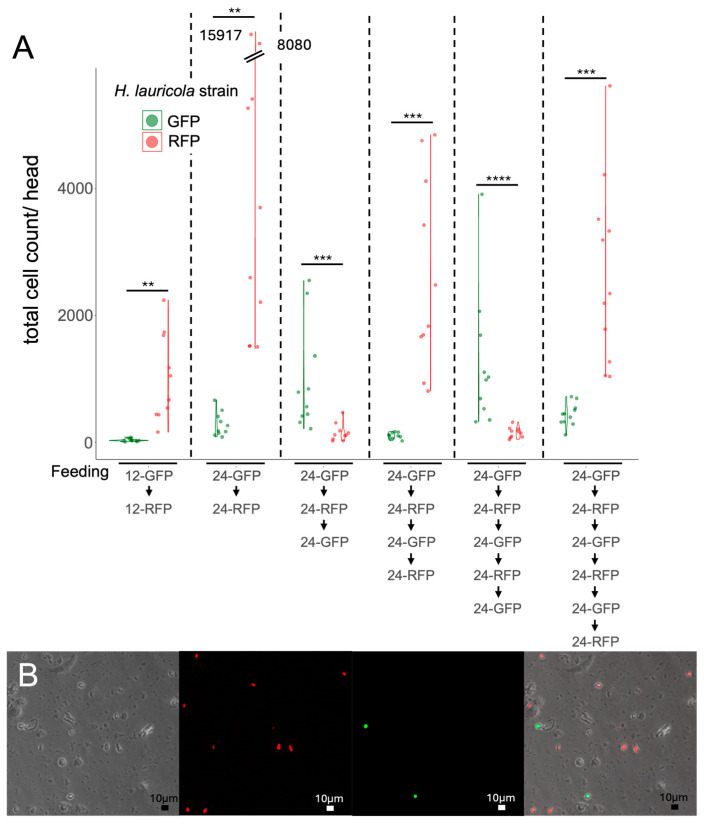
Quantification of turnover of *H. lauricola* cells in *X. affinis* mycangia by image cytometry using dual fluorescent signals. (**A**) Representative images were collected during the image cytometry run. Left: brightfield, left-center: red fluorescent (RFP) channel, right-center: green fluorescent (GFP) channel, right: overlay. (**B**) Quantification of image cytometry results. Asterisks indicate statistical differences with *p* values as follows: ** = 0.001 < *p* < 0.01, *** = 0.0001 < *p* < 0.001, **** = 0 < 0.0001.

Similarly to the experiments described above, the cell cytometry assay was used to examine the dynamic nature of the mycangia turnover. Newly emerged *X. affinis* beetles were first exposed to/allowed to feed on *H. lauricola^GFP^* for 12 h and then fed on RFP for 12 h (Figure 6A, leftmost data). To extend the “switching” assay, aposymbiotic beetles were first fed GFP for 24 h then RFP, with the sequential switching continued for 4, 5, and 6 d. Beetles fed the GFP strain for 12 h and then the RFP strain for 12 h showed almost complete switching to the RFP strain (1014 ± 217 RFP, 34 ± 6 GFP). In contrast, beetles fed GFP (for 24 h) and then RFP (24 h) showed low to moderate levels of the GFP cells (289 ± 60), with high levels of RFP cells (2966 ± 586). Beetles fed GFP-RFP-GFP showed moderately higher levels of GFP cells (986 ± 266), as compared to RFP (157 ± 44). Beetles fed GFP-RFP-GFP-RFP retained only low levels of the GFP cells (107 ± 15), with RFP overtaking (2655 ± 482). Beetles fed GFP-RFP-GFP-RFP-GFP showed a dramatic decrease in RFP (157 ± 26), with high levels of GFP cells (1269 ± 342). Beetles fed GFP-RFP-GFP-RFP-GFP-RFP retained low levels of GFP cells (439 ± 53), with high RFP cell levels (2687 ± 435). Fluorescent cell cytometry imaging of the microtiter plates containing the mycangial extracts allowed for the direct visualization of beetles fed in the switching assays, with both GFP- and RFP-expressing cells apparent (Figure 6B).

**Figure 7:**
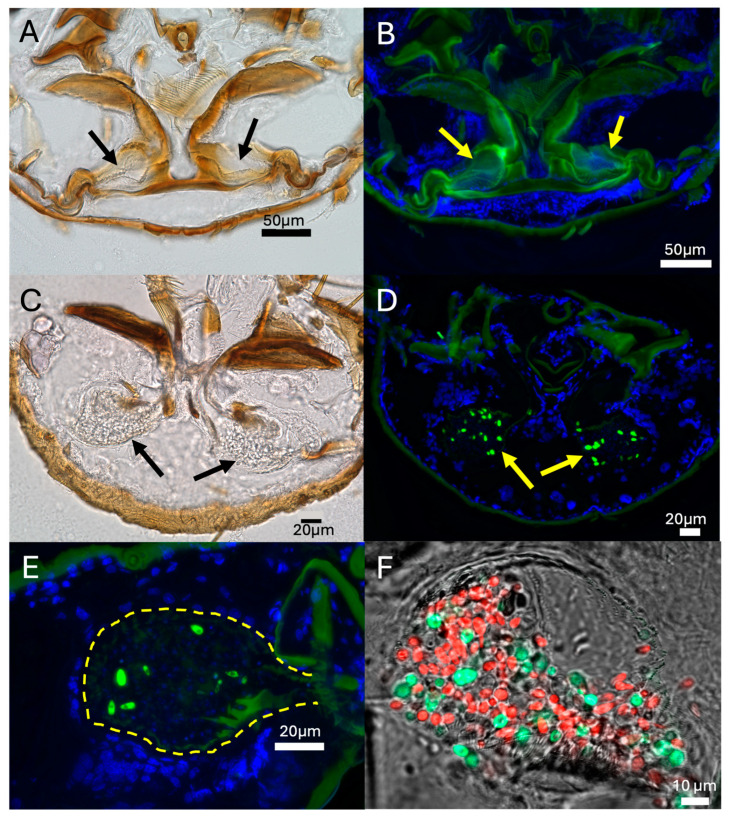
Characterization of fungal cells within mycangia and surrounding tissue during *H. lauricola* colonization of *X. affinis* mycangia by light microscopy (**A**,**C**) and fluorescence microscopy (**B**,**D**–**F**). For fluorescence images, GFP-expressing fungal cells, as well as autofluorescence from insect tissue are imaged in green, and nuclei stained with DAPI are imaged in blue. (**A**,**B**) Uncolonized aposymbiotic newly emerged *X. affinis* beetles showing no detectable fungal cells within the mycangia (**A**, arrows). (**C**,**D**) Beetle mycangia following colonization after 12 h of feeding (**C**, arrows, GFP-expressing *H. lauricola*). (**E**) High magnification image of (GFP-expressing) *H. lauricola* cells within mycangia after 6 h of feeding. (**F**) Representative image of a mixed population of green (GFP-) and red (RFP-) fluorescent protein expressing *H. lauricola* cells within the same mycangium occurring during alternating (switching) colonization assays.

To better define the mycangial organ, sections of uncolonized aposymbiotic beetles and those fed *H. lauricola^GFP^* for 12 h were examined via brightfield and fluorescent microscopy with samples counterstained with the nuclear dye DAPI (Figure 7A–D). Empty mycangial organs were visible in uncolonized beetles with the green autofluorescence of the surrounding mandibular structures. Brightfield and (GFP) fluorescent images of colonized mycangia revealed fungal cells within the two mycangial organs. Insect cells, as seen by DAPI staining, could be seen around the contours of the mycangia as well as throughout the head. Fungal cells within the mycangia could be seen within 6 h of feeding (Figure 7E), and mixed populations of *H. lauricola^RFP^* and *H. lauricola^GFP^* could be seen for cells fed *H. lauricola^GFP^* for 12 h and then switched to *H. lauricola^RFP^* for 12 h (Figure 7F).

## 4. Discussion

The nature and diversity of mutualistic associations between fungi and animals remains largely understudied. Ambrosia beetles are polyphyletic and are derived from divergent lineages of beetles with equally divergent fungal partners [3,12,35]. Similarly, “mycangia” represent the different “organs” evolved by ambrosia beetles to house and transport their respective fungal partners that vary greatly in structure, location on/within the insect body, and mechanisms of development and selection [22,26,36,37]. Characterization of the contents of mycangia has typically relied on isolation of fungal colonies on plates, and/or the application of metagenomic techniques [6,38,39,40]. More recently, a systematic characterization of the dynamics of mycangial colonization demonstrating host switching from the invasive (to the US) beetle species *X. glabratus*, which carried with it the (invasive) laurel wilt fungal pathogen, *H. lauricola*, to *X. affinis* beetles has been reported [17]. These data also demonstrated that the *H. lauricola* colonization of *X. affinis* is stable, maintained even during starvation, and shows rapid turnover within the early period of mycangial colonization (up to 3 d). These and other data reported for *H. lauricola* isolated from environmental samples and laboratory-reared *Xyleborus* beetles (*X. affinis*, *X. bispinatus*, *X. volvulus*, and *X. glabratus*) have all relied on plating of mycangial contents and CFU calculations [16,41,42,43]. However, CFU counting could involve systematic biases since: (i) fungal cells may grow as hyphae (or some other multicellular form) and, therefore, CFU counts would underestimate actual fungal biomass, and (ii) CFU counts would only capture viable cells, with the potential of the existence of a pool of non-viable cells in the mycangia [44]. In addition, CFU counting can be time-consuming (requires waiting for the colonies to grow), and factors such as dilutions, number of replicates, and/or extraction protocols can lead to errors. To overcome these issues, we applied a simple image cell cytometry approach to examining mycangial contents. By using cells expressing a fluorescent reporter (GFP or RFP), the method can identify and discriminate fungal cells from insect or other tissues/cells in a facile manner. Results from the cell cytometry approach agreed with CFU counting, although, in general, the image cell cytometry method yielded a larger range of counts at the higher end, i.e., data points with >10,000 cells counted, as compared to the CFU method.

Using both CFU counting and the image cell cytometry and our fluorescent reporter strains (*H. lauricola^RFP^* and *H. lauricola^GFP^*), we sought to address how long-term occupancy (24 h), followed by switching to another strain (for 24) and then continuing this “switching” over a 7 d time course affects mycangial contents. These data confirmed that even such long-term colonization and switching results in dynamic turnover, with mycangial contents following the introduction of “fresh” fungal cells, albeit some of the fungal cells from the previous feeding period always remained. Both CFU and cell cytometry data were in good agreement, except for the last switching timepoint (7 d), in which the CFU data showed significant retention of the previous fed (in this case, *H. lauricola^GFP^* strain after switching to the *H. lauricola^RFP^* strain). In contrast, the cell cytometry showed very low levels of *H. lauricola^GFP^*, which was mainly replaced by *H. lauricola^RFP^* cells. The most likely explanation may be a combination of higher error in the CFU coupled with the large variation seen in the data in general. Based upon these and our previous data [17], we now consider this to be an intrinsic aspect of mycangial colonization. Reasons for this variation can include: (i) an aspect of the dynamic turnover, i.e., because of cells entering and leaving the mycangia, (ii) a difference in the nutritional state of the beetle, which may affect feeding and/or mycangial occupancy, and/or (iii) differences in the nutritional state of the fungus.

With respect to the nutritional state of the fungus, mycangial colonization using cells growing on standard media (PDA, “medium level” nutritional substrate) showed robust colonization of the mycangia. Growth on the CZA (minimal media containing sucrose as the sole source of carbon) showed similar results. Intriguingly, growth on the rich media (SDA and SDA + yeast extract) resulted in poor mycangial colonization. Two main factors could account for these results. In rich media, there would be significantly more hyphae and mycelium, which may be poor candidates for sequestering into the mycangia. Second, the tree gallery (where the fungus is grown) is likely a nutritionally poor substrate more similar to PDA/CZA and, hence, may favor the production of cells more amenable to mycangial colonization. Our data also show significant differences in colonization by different *Xyleborus* partner fungi. For *X. affinis*, both the (invasive) *H. lauricola* and the (indigenous) *R. arxii* were good mycangial colonizers. Intriguingly, the (indigenous) *H. aguacate* was apparently a poor partner for *X. affinis*. The reasons for this are unclear and an additional wider survey of fungal partners can help shed light on this issue. To confirm specificity of the relationship, we tested the non-ambrosia fungus and the plant pathogen, *M. oryzae*, and demonstrated that this fungus is neither eaten nor able to colonize the mycangia. These data also indicate that ambrosia beetles are not likely to vector any non-ambrosia fungi (plant pathogens) except accidently. We further show that the related (indigenous) *X. ferrugineus* ambrosia beetle does not appear to be a good host for *H. lauricola*. Again, these data suggest mechanisms of specificity that remain obscure and indicate the need for examining wider pairings between ambrosia beetles and their fungal partners.

Fungal-animal mutualisms are important ecological, evolutionary, and even pathogenic systems that remain understudied. The ambrosia beetle–fungal partner symbioses are a unique model that can provide insights into the dynamics of co-evolution and its consequences on host and partner development. Significant questions remain regarding how specificity in the mycangia is achieved, the nature of the fungal–host interactions, i.e., potential for nutrient exchange, and the mechanism by which (the pre-oral) mycangia in *Xyleborus* beetles function, i.e., how do the fungal cells enter and exit? Our data provide a framework for further exploring such questions via quantitative and cellular approaches and reveals new insights into the fidelity of associations between closely related species of ambrosia beetles and their indigenous and invasive fungal partners. Furthermore, we present new methodologies for studying the interactions between ambrosia fungi and their beetle hosts within mycangia, mutualistic organs essential for widespread and ecologically consequential symbioses. Coupling these efforts with genetic investigations will help undercover the physiological and molecular determinants that underlie such fungal-animal mutualisms.

## Data Availability

The original contributions presented in this study are included in the article. Further inquiries can be directed to the corresponding authors.
